# Assessment of global DNA methylation in the first trimester fetal tissues exposed to maternal cigarette smoking

**DOI:** 10.1186/s13148-016-0296-0

**Published:** 2016-11-25

**Authors:** Svetlana Fa, Trine Vilsbøll Larsen, Katrine Bilde, Tina F. Daugaard, Emil H. Ernst, Rasmus H. Olesen, Linn S. Mamsen, Erik Ernst, Agnete Larsen, Anders L. Nielsen

**Affiliations:** 1 Department of Biomedicine, Aarhus University, Bartholin building, DK-8000 Aarhus C, Denmark; 2 Faculty of Sciences, University of Novi Sad, Novi Sad, Serbia; 3 Laboratory of Reproductive Biology, The Juliane Marie Centre for Women, Children and Reproduction, University Hospital of Copenhagen, University of Copenhagen, Copenhagen, Denmark; 4 Department of Obstetrics and Gynecology, University Hospital of Aarhus, Skejby Sygehus, Aarhus, Denmark

**Keywords:** Epigenetics, DNA methylation, Smoking, Xenochemicals, Toxicology

## Abstract

**Aims:**

Maternal cigarette smoking during pregnancy increases the risk of negative health consequences for the exposed child. Epigenetic mechanisms constitute a likely link between the prenatal exposure to maternal cigarette smoking and the increased risk in later life for diverse pathologies. Maternal smoking induces gene-specific DNA methylation alterations as well as global DNA hypermethylation in the term placentas and hypomethylation in the cord blood. Early pregnancy represents a developmental time where the fetal epigenome is remodeled and accordingly can be expected to be highly prone to exposures with an epigenetic impact. We have assessed the influence of maternal cigarette smoking during the first trimester for fetal global DNA methylation.

**Methods and results:**

We analyzed the human fetal intestines and livers as well as the placentas from the first trimester pregnancies. Global DNA methylation levels were quantified with ELISA using a methylcytosine antibody as well as with the bisulfite pyrosequencing of surrogate markers for global methylation status, *LINE*-*1*, and *AluYb8*. We identified gender-specific differences in global DNA methylation levels, but no significant DNA methylation changes in exposure responses to the first trimester maternal cigarette smoking.

**Conclusions:**

Acknowledging that only examining subsets of global DNA methylation markers and fetal sample availability represents possible limitations for the analyses, our presented results indicate that the first trimester maternal cigarette smoking is not manifested in immediate aberrations of fetal global DNA methylation.

**Electronic supplementary material:**

The online version of this article (doi:10.1186/s13148-016-0296-0) contains supplementary material, which is available to authorized users.

## Introduction

Prenatal exposure to maternal cigarette smoking (PEMCS) represents a fetal exposure with consequences for the birth weight and delivery term [1, 2]. PEMCS also predisposes individuals for diseases later in life [3–5]. This includes reduced pulmonary function and increased asthmatic symptoms in childhood [6–9], changes in children’s neurodevelopment and behavior [10–12], an increased incidence of childhood obesity and metabolic disorders [13–15], and reduced cardiovascular health among children [16–19]. In the Western world, approximately 25% of women of fertile age are smoking cigarettes and 7% continue smoking throughout pregnancy [4]. Epigenetics, at least in part, may explain the connection between PEMCS and increased disease risk later in life [3, 20]. Epigenetics can mechanistically describe the regulation of cellular gene expression in response to a given environment, with epigenetics functioning through short and long non-coding RNA (ncRNA), chromatin remodeling, histone modification, and DNA methylation. DNA methylation patterns can be dynamic, display the cell type and tissue specificity, and change upon environmental exposure [21–23]. The DNA methylation profile in the zygote is reprogrammed during the cleavage phase with massive de novo DNA methylation following the implantation phase [24, 25]. Stringent-controlled dynamics for removing DNA methylation and de novo DNA methylation is essential for correct development, with early embryogenesis representing one critical window in which environmental factors can influence DNA methylation in offspring [22, 23]. PEMCS is shown to induce quantitative alterations in position-specific CpG methylation in the placenta and blood from newborns, and DNA methylation changes can be maintained into adulthood [3, 20, 26–34]. The latter are exemplified in the longitudinal studies by Richmond et al. and Lee et al., in which the researchers collected peripheral blood samples and examined the methylation status of CpG sites manifesting PEMCS-induced DNA methylation changes in the cord blood at birth [30, 34]. The longitudinal analyses at age 17 years showed persistently changed patterns of DNA methylation for CpG sites in *AHRR* (cg05575921), *MYO1G* (cg22132788), *CYP1A1* (cg09935388), and *CNTNAP2* (cg25949550), whereas the reversibility of DNA methylation was observed for CpG sites in *GFI1* (cg09935388), *KLF13* (cg26146569), and *ATP9A* (cg07339236) [30, 34].

Similar to locus-specific DNA methylation changes, global DNA methylation changes also represent a biodosimeter of lifelong environmental exposures [35]. The consequence of PEMCS for global methylation was addressed by Wilhelm-Benartzi et al., who in placenta tissue examined both gene-associated loci and *long interspersed nuclear element*-*1* (*LINE*-*1*) and *AluYb8* repetitive elements [36]. *LINE* and *Alu* repeat elements act as surrogate markers for global DNA methylation measurements [36, 37]. In the term placentas, the DNA methylation level of *AluYb8* was significantly higher among infants prenatally exposed to cigarette smoke, whereas no significant DNA methylation changes were observed for *LINE*-*1* and gene-associated loci [36]. Another study of term placenta samples also failed to find any significant association between *LINE*-*1* DNA methylation and PEMCS [38] but, notably, gene-associated CpG site-specific DNA methylation alterations have been described in the placenta [28, 33, 39, 40]. In the cord blood, using methyl-specific ELISA-based methodology, global DNA hypomethylation was observed in newborns from PEMCS and second-hand smoking exposure [41]. For *LINE*-*1,* no significant change in DNA methylation in the cord blood was observed between cigarette smoke-exposed and non-exposed children, but an association between *LINE*-*1* DNA methylation status and birth weight was present [38]. In buccal cells from children with PEMCS, *AluYb8* hypomethylation was observed, whereas in *LINE*-*1* DNA methylation was not significantly affected [42].

Even if PEMCS is described to induce global alterations in DNA methylation present at the time of birth and such changes, at least to some extent, can be maintained into later life, to our knowledge no descriptions are present of the timing for the developmental onset of PEMCS-mediated global DNA methylation changes. Accordingly, we examined whether PEMCS-induced global DNA methylation changes are manifested already during early fetal development, a period expected to be particularly prone to exposure-induced epigenetic alterations.

## Materials and methods

### Ethics statement and sample collection

The fetal tissues were obtained with informed consent from women seeking a legal (<gestation week 12) abortion in a regional hospital within Region Midtjylland, Denmark. The study was approved by The Danish National Committee on Health Research Ethics (approval no. KF (01)258206), and all the experiments were performed in accordance with the Helsinki Declaration. There was no change in treatment or care associated with recruitment to the study. All personal identification data are anonymized. Information concerning smoking habits was obtained. Cotinine concentrations were previously measured in maternal serum and fetal organs for a similar sample cohort, and the observed correspondence between reported smoking status and cotinine concentration supported the reliability of the women’s self-reported smoking habits [43]. Fetal tissue (6 to 12 weeks of pregnancy) was surgically removed from the uterus according to routine procedures. The age of the fetuses was determined by ultrasound examination prior to the procedure. Immediately after the surgical procedure, placental and fetal tissues were rinsed in sterile isotonic saline and carefully dissected under a stereomicroscope. All fetuses appeared morphologically normal, and no disease was suspected prior to the procedure. The small intestine, liver, and placenta samples were isolated, rinsed in sterile isotonic saline and placed in separate tubes containing RNA-later (Ambion, Inc., Austin, TX, USA). The samples were stored for 2–4 hours at room temperature and then frozen at −20 °C.

Tissue biopsies were snap frozen in RNA-later (Ambion, Inc., Austin, TX, USA). The sample collection was continuously expanded throughout the study period, and samples representing each tissue and experimental assay are not systematically the same. The numbers of samples for each tissue (*N*), subdivided into female (f) and male (m) samples, or smoking (S) and non-smoking (NS) samples, were *N* = 40, with 19 f and 21 m and 17 S and 23 NS, for the placenta; *N* = 33, with 14 f and 19 m and 18 S and 15 NS, for the liver; and *N* = 21, with 15 f and 6 m and 12 S and 9 NS, for the small intestine. More details on the samples used for each particular experimental assay including information on fetal gender, age, and smoking status of the mothers are described in Additional file 1: Table S1 as well as in the figure legends for the given experimental settings. Differences in the age distribution for males and females, as well as for smoking-exposed or non-smoking-exposed groups, were, if present, encountered in subsequent data analyses.

### DNA extraction and gender determination

DNA was extracted using the MasterPure™ Complete DNA and RNA Purification Kit (Epicentre, Madison, WI, USA). DNA quantities were measured using a Nanodrop spectrophotometer (Thermo Scientific, Wilmington, USA). Gender was determined by pyrosequencing, as previously described [44].

### ELISA-based global DNA methylation quantification

The percentage of methylation in total DNA was determined by measuring 5-methylcytosine (5-mC) using a 5-mC DNA ELISA kit (Zymo Research Corp, Orange, CA, USA) that features a unique anti-5-mC monoclonal antibody that is both sensitive and specific for 5-mC. Assays were performed according to the manufacturer’s instructions with the modified standard curve. The percentages of prepared methylated DNA standards were 5, 1.66, 0.55, and 0.185%. The amount of total DNA used was 100 ng. Logarithmic second-order regression was used to calculate the results. Since the positive and negative controls used to prepare the standard curve consist of *Escherichia coli* gDNA, the obtained results were multiplied by 5.68 to correct for fold differences in the density of CpGs between *E. coli* and humans following the recommendations from the manufacturer.

### Bisulfite conversion and pyrosequencing for global DNA methylation analyses

A total of 1.2 μg of the small intestine and 2 μg of the placenta and liver genomic DNA was bisulfite-converted using the EpiTect Bisulfite Kit (Qiagen, Hilden, Germany) according to the manufacturer’s instructions. Specific genomic DNA sites were amplified by PCR using 5 μl of bisulfite-converted genomic DNA as templates. PCR reactions were performed using PyroMark PCR kit (Qiagen) according to the manufacturer’s instructions. Pyrosequencing for quantitative DNA methylation analyses was performed using the PyroMark Q24 Advanced system (Qiagen). *LINE*-*1* amplification and pyrosequencing was performed using a predesigned assay PyroMark Q24 CpG LINE-1 (Qiagen). *AluYb8* amplification and pyrosequencing was performed as previously described [37].

### Statistics

An unpaired *t* test was used to compare two sample groups, and a comparison between multiple groups was done using two-way (gender and smoking exposure) analysis of variance (two-way ANOVA) using the GraphPad Prism (GraphPad Software, La Jolla, CA, USA). Analysis of covariance (ANCOVA) was used to examine the interaction between smoking, gender, and age, where age was treated as a continuous exploratory variable and smoking status and gender were treated as factors. ANCOVA was carried out using R version 3.3.1, and the outcomes are presented in Table 1. The normal distribution of data was investigated by QQ-plot, whereas the linearity and the homoscedasticity of residuals were assessed by inspecting a plot of the residuals against the explanatory variables. We did not observe any violation of the assumptions for the analyses. In all analyses, values of *p* < 0.05 were considered statistically significant.

**Table 1:** ANCOVA for the effects of fetal age, gender, and PEMCS for DNA methylation levels in the first trimester placentas, fetal livers, and small intestines

Tissue	Assay	Variable	*F* value	*P* value
Placenta	5-mC ELISA	Age	8.567	0.008*
		Gender	8.434	0.008*
		PEMCS	0.704	0.411
	*AluYb8*	Age	0.059	0.811
		Gender	4.527	0.042*
		PEMCS	2.958	0.096
	*LINE*-*1*	Age	0.376	0.544
		Gender	0.013	0.909
		PEMCS	1.017	0.320
Liver	5-mC ELISA	Age	1.214	0.286
		Gender	0.191	0.667
		PEMCS	0.001	0.977
	*AluYb8*	Age	3.190	0.085
		Gender	0.004	0.953
		PEMCS	0.106	0.747
	*LINE*-*1*	Age	0.263	0.612
		Gender	8.779	0.006*
		PEMCS	0.672	0.419
Small intestine	5-mC ELISA	Age	3.576	0.078
		Gender	0.445	0.515
		PEMCS	0.147	0.707
	*AluYb8*	Age	0.006	0.942
		Gender	1.417	0.250
		PEMCS	0.121	0.733
	*LINE*-*1*	Age	0.327	0.575
		Gender	0.568	0.462
		PEMCS	1.783	0.201

Analyses were done with age as a continuous variable and gender and PEMCS as factors

*Indicates statistical significance, *p* < 0.05

## Results

### Placenta global DNA methylation levels and PEMCS

To characterize the impact of PEMCS for global DNA methylation on fetuses and placentas, we sampled the human placentas, fetal livers, and fetal small intestines representing days 44 to 82 post-gestation. Gender was determined by pyrosequencing a polymorphic region of the amelogenin gene, as previously described [45]. Smoking status was assigned to mothers with self-reported daily smoking (approximately 90% of the smoking mothers reported smoking 6–20 cigarettes per day). In addition, information regarding exposure to passive smoking was obtained. Whereas cigarette smoking mothers also reported exposure to passive smoking, this additional exposure was not reported to be present among the non-smoking mothers. A description of the gender, age, and smoking status of the study samples is presented in Additional file 1: Table S1.

**Fig. 1:**
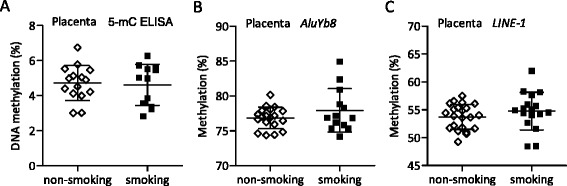
Global DNA methylation in placentas. DNA methylation determined by ELISA using **a** 5-mC-specific antibody (non-smoking group *N* = 15 and smoking group *N* = 12). Data represent the means of two experiments. The average DNA methylation percentage for **b** five consecutive *AluYb8* CpG sites (non-smoking group *N* = 19 and smoking group *N* = 13) and **c** three consecutive *LINE*-*1* CpG sites (non-smoking group *N =* 22 and smoking group *N* = 17) were determined by pyrosequencing bisulfite-converted DNA. Data represent the means of two runs, except for samples that differed more than 5% in methylation levels within the duplicates. For such samples, a third run was performed and the presented data represent the means of the three runs. Mean methylation percentages ± SD are shown. Unpaired *t* test was used to compare two groups. A *p* value < 0.05 was considered statistically significant

To address whether PEMCS manifests global DNA methylation alterations during the first trimester in the placenta, we first performed an ELISA methylcytosine antibody-based measurement of global DNA methylation (5-mC ELISA). No statistically significant effects of PEMCS were observed for the global DNA methylation level in the placenta (Fig. 1a). Gender-specific DNA methylation quantitative effects of PEMCS have been described [46–48], and accordingly, a two-way ANOVA was performed to investigate the impact of PEMCS and gender on DNA methylation levels. We observed that lower methylation levels were present in the placenta from female pregnancies than male pregnancies (*p* = 0.004, two-way ANOVA) (Additional file 2: Figure S1A). To investigate whether fetal age affects DNA methylation levels in the placenta, we performed ANCOVA with age, gender, and PEMCS status as variables (Table 1). This analysis identified a statistically significant increase in global DNA methylation with age (*p* = 0.008) (Table 1). Altogether, the ELISA analyses showed that global DNA methylation levels in the placenta are affected by fetal age and gender but not by PEMCS.

The average DNA methylation level for repetitive *AluYb8* elements represents an estimate for the global DNA methylation level [37]. By measuring *AluYb8* DNA methylation levels in term placenta, Wilhelm-Benartzi et al. identified that PEMCS resulted in hypermethylation (66.2% in PEMCS exposed versus 64.8% in non-exposed placenta tissue) [36]. We analyzed our samples with an *AluYb8* bisulfite pyrosequencing assay similar to the assay used by Wilhelm-Benartzi et al. [36]. The *AluYb8* bisulfite pyrosequencing assay measures the DNA methylation status of five consecutive CpG sites. For each tested DNA sample, we determined the average DNA methylation level for these five CpG sites. A comparison between smoking-exposed and non-exposed samples revealed that PEMCS did not induce alterations in *AluYb8* DNA methylation (Fig. 1b). We next questioned whether differences in DNA methylation levels existed between smoking-exposed and non-exposed samples at individual *AluYb8* CpG sites. We detected no PEMCS-induced changes in DNA methylation levels for any of the five CpG sites (Additional file 2: Figure S1B). Two-way ANOVA using the DNA methylation data for the average *AluYb8* DNA methylation level for each sample identified a lower *AluYb8* DNA methylation level in females than males (*p* = 0.036, two-way ANOVA) (Additional file 2: Figure S1C). We note that *AluYb8* methylation levels in term placentas previously were described to be gender dependent [36]. A subsequent ANCOVA revealed no impact of the fetal age on *AluYb8* DNA methylation in placentas (Table 1). Similar to *AluYb8*, the average DNA methylation level for repetitive *LINE*-*1* elements represents an estimate for the global DNA methylation level [37]. We analyzed our samples with a *LINE*-*1* bisulfite pyrosequencing assay. The used *LINE*-*1* bisulfite pyrosequencing assay measures DNA methylation levels for three consecutive CpG sites. For each tested DNA sample, we determined the average methylation level of all three CpG sites. A comparison between smoking-exposed and non-exposed samples revealed that PEMCS did not induce alterations in *LINE*-*1* DNA methylation (Fig. 1c). We next questioned whether differences in DNA methylation levels existed between smoking-exposed and non-exposed samples at individual *LINE*-*1* CpG sites. We detected no PEMCS-induced changes in DNA methylation levels for any of the three CpG sites (Additional file 2: Figure S1D). Moreover, two-way ANOVA did not detect gender differences in *LINE*-*1* DNA methylation levels (Additional file 2: Figure S1E), and ANCOVA revealed no association between *LINE*-*1* DNA methylation levels and fetal age (Table 1). From the described analyses of surrogate repetitive element markers, we conclude that PEMCS in the first trimester is not manifested in placenta global DNA methylation changes and no interaction is present between PEMCS, gender, and age (data not shown).

### Global methylation levels in fetal livers and small intestines and PEMCS

**Fig. 2:**
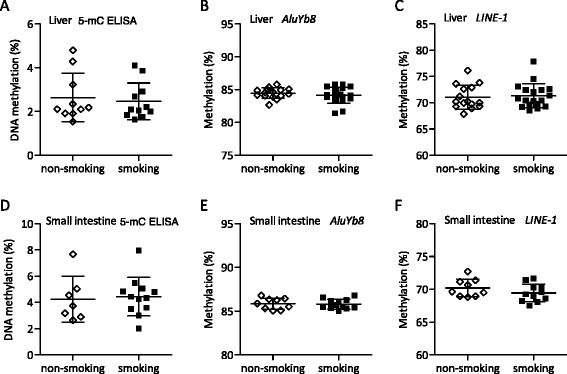
Global DNA methylation in fetal livers and small intestines. **a** DNA methylation in the fetal liver was determined by ELISA using 5-mC-specific antibody (non-smoking group *N* = 10 and smoking group *N* = 11). Data represent the means of three experiments. **b**–**c** Percentage of methylation of *AluYb8* and *LINE*-*1* in the fetal liver was determined by pyrosequencing bisulfite-converted DNA (non-smoking group *N =* 15 and smoking group *N* = 18). **d** DNA methylation in the fetal small intestine was determined by ELISA using 5-mC-specific antibody (non-smoking group *N* = 7 and smoking group *N* = 12). Data represent the means of 2 experiments. **e**–**f** Percentage of methylation of *AluYb8* (non-smoking group *N* = 9 and smoking group *N* = 12) and *LINE*-*1* (non-smoking group *N =* 9 and smoking group *N* = 11) was determined by pyrosequencing bisulfite-converted DNA. Percentages of methylation were calculated, analyzed and displayed as described in the legend for Fig. 1

We next questioned whether global changes in DNA methylation were present in the fetus due to the first trimester PEMCS. We first examined global quantitative DNA methylation levels in the fetal livers, which represent a key metabolic target tissue. In a 5-mC ELISA-based DNA methylation analysis, we identified no significant differences caused by PEMCS (Fig. 2a). Two-way ANOVA revealed a similar level of DNA methylation in males and females (Additional file 3: Figure S2A) and no impact of fetal age for the global DNA methylation level in the fetal livers were detected by ANCOVA (Table 1). We next addressed *AluYb8* DNA methylation with the same bisulfite pyrosequencing assay used for the placenta analysis*.* PEMCS was not identified to have an impact on the average DNA methylation level of the five *AluYb8* CpG sites tested (Fig. 2b). DNA methylation levels for the individual *AluYb8* CpG sites also were unaffected by PEMCS (Additional file 3: Figure S2B). Gender was not identified to have an impact on *AluYb8* DNA methylation (Additional file 3: Figure S2C). *AluYb8* DNA methylation levels were not affected by the age of the fetal liver samples (Table 1, ANCOVA). *LINE*-*1* DNA methylation levels were next measured with the same bisulfite pyrosequencing assay used for placenta samples. No significant effects of PEMCS were observed for *LINE*-*1* methylation, either for the average DNA methylation level for the three *LINE*-*1* CpG sites tested or for each of the three individual CpG sites (Fig. 2c, Additional file 3: Figure S2D). We detected significantly lower *LINE*-*1* DNA methylation levels in females compared to that in males (*p* = 0.009, two-way ANOVA) (Additional file 3: Figure S2E). *LINE*-*1* DNA methylation levels were not affected by the age of the fetal liver samples (Table 1, ANCOVA).

To investigate another fetal tissue, we also performed DNA methylation analyses of the fetal small intestine. 5-mC ELISA analysis showed no significant changes in the small intestine global DNA methylation levels from PEMCS (Fig. 2d). We also did not detect gender or age effects for the global DNA methylation levels in the fetal small intestines (Table 1 and Additional file 4: Figure S3A). In accordance, bisulfite pyrosequencing of *AluYb8* and *LINE*-*1* showed no significant effects for the DNA methylation levels in the small intestines (Fig. 2e and f, Additional file 4: Figure S3B and S3D). Gender and age had no significant effect on the *AluYb8* and *LINE*-*1* DNA methylation levels in the small intestines (Table 1 and Additional file 4: Figure S3C and S3E). From the presented results based on the analyses of surrogate markers for global DNA methylation status, we conclude that, at least in the hereby examined sample cohort, the first trimester PEMCS does not affect the global DNA methylation level in fetal small intestines and livers.

## Discussion

In the presented study, we have examined whether PEMCS results in global DNA methylation changes in the first trimester placentas and fetal livers and small intestines. In term placentas and umbilical cord blood, as well as in the peripheral blood and buccal epithelium tissue of children, PEMCS-induced differences in global DNA methylation have previously been described [36, 41, 42]. However, for tissue samples representing early fetal development, to our knowledge, results addressing maternal smoking and global DNA methylation alterations, do not exist, and this lack of information reflects the fact that relevant experimental samples are rarely obtainable. In our analyses examining the first trimester placentas and fetal livers and small intestines, we did not identify significant PEMCS-induced alterations in global DNA methylation levels. This indicates either higher sensitivity for a DNA methylation response later in fetal development or the requirement of a long-term dose-effect response before smoking-mediated DNA methylation alterations will be manifested at birth and in later life [34, 49]. Notably, an immediate manifestation of gene-specific DNA methylation alterations from maternal smoking in the first trimester was shown in the study by Chhabra et al. [39]. Methylation array analyses identified gene-specific DNA methylation alterations associated with nicotine exposure, but only a few of these changes were similar to the DNA methylation changes described to be present at birth, in childhood, or in adulthood due to PEMCS [39]. This observation, as well as our results for global DNA methylation levels, is in contrast to the general assumption that early pregnancy represents the most sensitive period for the lifelong manifestation of environmental-induced epigenetic changes. Chhabra et al.’s [39] observation of gene-specific DNA methylation alterations in the first trimester tissue due to maternal smoking, while global DNA methylation levels were not affected, could reflect the remodeling of specific signaling pathways by maternal cigarette smoking at this particular developmental time. We note the existence of developmental consequences of the first trimester smoking exposure since the number of germ cells is significantly reduced in the first trimester fetuses exposed to maternal cigarette smoke [43].

The relative low number of available fetal samples represents a limitation for the current study since it could prevent the detection of subtle methylation differences; however, we were unable to increase the number of samples given the limited availability of the human samples for our analyses. A possible confounder in studies of the effect of PEMCS is the reliability of self-reported smoking status, as well as exposure to household smoking, which has been found to cause the same DNA methylation effects in newborns as PEMCS [27, 41]. Most women are aware of the fact that smoking during pregnancy can be harmful to their child and may therefore understate their smoking in the mother-child analyses [50–52]. However, as the women included in the present study sought the active termination of their pregnancies, their willingness to report smoking could be more reliable. In support of this, measurements of cotinine levels in similar collected samples from women undergoing the legal termination of their pregnancies have verified the reliability of the women’s self-reported smoking habits [43]. In addition, we note that all the non-smoking women in the study reported an absence of exposure to second-hand smoking, whereas nearly all smoking women reported additional exposure to second-hand smoking. Finally, transgenerational smoking effects as well as the preconception smoking of the father and the mother have potential consequences for the fetal epigenome and can confound the methylation levels measured in the non-exposed samples [20].

## Conclusion

Smoking cessation prior to the second trimester of pregnancy seems to have many of the same health benefits as smoking cessation before pregnancy or never having smoked [2, 13, 53, 54]. To the best of our knowledge, the presented data are the first to show that global fetal DNA methylation is not significantly changed in organs from the first trimester PEMCS. Obviously, site-specific CpG methylation alterations from PEMCS with potential devastating health consequences can already be manifested from the first trimester smoking exposure, but not elucidated in the present analyses of global alterations. This was exemplified by Chhabra et al., who showed an association between gene-specific DNA methylation changes and nicotine exposure in the first trimester lung and placenta [39]. The current clinical guidelines highlight the importance of the cessation of cigarette smoking before or early in the pregnancy. It is important to stress that epidemiological data and gene-specific DNA methylation data still invariably support reinforced attempts of the immediate smoking cessation for women still smoking at the time of pregnancy recognition to improve prenatal care.

## Additional files

Additional file 1: Table S1. Description of fetal and placenta samples. The number of samples (*N*) and fetal age in days are illustrated. Samples were assigned to the smoking-exposed group if mothers smoked one or more cigarettes per day (approximately 90% of the smoking mothers were smoking 6–20 cigarettes per day). Exposure to passive smoking was not reported among the non-smoking mothers, whereas among the smoking mothers most also reported exposure to passive smoking. Unpaired *t* test was used to determine whether there is a statistically significant age difference between the non-smoking and smoking groups and between females and males used for a particular assay (*LINE*-*1*, *AluYb8,* and 5-mC ELISA). *Indicates statistical significance, *p* < 0.05. (DOCX 17 kb)
Additional file 2: Figure S1. DNA methylation in placentas. **a** DNA methylation in female and male fetal placentas determined by the Elisa method using 5-mC-specific antibody (non-smoking female group *N* = 6, non-smoking male group *N* = 9, smoking female group *N* = 7, and smoking male group *N* = 5). Data represent the mean of two experiments. **b** Percentage of *AluYb8* DNA methylation at five consecutive CpG sites (non-smoking group *N* = 19 and smoking group *N* = 13). **c** *AluYb8* DNA methylation in males and females (non-smoking female group *N* = 7, non-smoking male group *N* = 12, smoking female group *N* = 8, and smoking male group *N* = 5). **d** Percentage of *LINE*-*1* DNA methylation at three consecutive CpG sites (non-smoking group *N =* 22 and smoking group *N* = 17). **e** *LINE*-*1* DNA methylation in males and females (non-smoking female group *N* = 9, non-smoking male group *N* = 13, smoking female group *N* = 9, and smoking male group *N* = 8). Percentages of methylation were calculated and displayed as described in the legend for Fig. 1. Unpaired *t* test was used to compare non-smoking and smoking exposed group, while two-way ANOVA was used to compare combined effects of smoke exposure and gender. *Indicates statistical significance, *p* < 0.05. (PDF 559 kb)
Additional file 3: Figure S2. DNA methylation in fetal livers. **a** DNA methylation in female and male fetal livers determined by the Elisa method using 5-mC-specific antibody (non-smoking female group *N* = 4, non-smoking male group *N* = 6, smoking female group *N* = 6, and smoking male group *N* = 5). Data represent the mean of three experiments. **b** Percentage of *AluYb8* DNA methylation at 5 consecutive CpG sites (non-smoking group *N =* 15 and smoking group *N* = 18). **c** *AluYb8* DNA methylation in males and females (non-smoking female group *N* = 6, non-smoking male group *N* = 9, smoking female group *N* = 8, and smoking male group *N* = 10). **d** Percentage of *LINE*-*1* DNA methylation at three consecutive CpG sites (non-smoking group *N* = 15 and smoking group *N* = 18). **e** *LINE*-*1* DNA methylation in males and females (non-smoking female group *N* = 6, non-smoking male group *N* = 9, smoking female group *N* = 8, and smoking male group *N* = 10). Percentages of methylation were calculated and displayed as described in the legend for Fig. 1. Unpaired* t* test was used to compare non-smoking and smoking exposed group, while two-way ANOVA was used to compare combined effects of smoke exposure and gender. *Indicates statistical significance, *p* < 0.05. (PDF 539 kb)
Additional file 4: Figure S3. DNA methylation in fetal small intestines. **a** DNA methylation in female and male fetal small intestines determined by the Elisa method using 5-mC-specific antibody (non-smoking female group *N* = 5, non-smoking male group *N* = 2, smoking female group *N* = 8, and smoking male group *N* = 4). Data represent the mean of two experiments. **b** Percentage of *AluYb8* methylation at 5 consecutive CpG sites (non-smoking group *N =* 9 and smoking group *N* = 12). **c** *AluYb8* DNA methylation in males and females (non-smoking female group *N* = 7, non-smoking male group *N* = 2, smoking female group *N* = 8, and smoking male group *N* = 4). **d** Percentage of *LINE*-*1* DNA methylation at three consecutive CpG sites (non-smoking group *N =* 9 and smoking group *N* = 11). **e** *LINE*-*1* DNA methylation in males and females (non-smoking female group *N* = 7, non-smoking male group *N* = 2, smoking female group *N* = 7, and smoking male group *N* = 4). Percentages of methylation were calculated and displayed as described in the legend for Fig. 1. Unpaired *t* test was used to compare non-smoking and smoking exposed group, while two-way ANOVA was used to compare combined effects of smoke exposure and gender. (PDF 529 kb)

## Abbreviations

5-mC: 5-Methylcytosine
*LINE*-*1*: Long interspersed nuclear element-1
PEMCS: Prenatal exposure to maternal cigarette smoking
