# Supplementary material for: Assessment of global DNA methylation in the first trimester fetal tissues exposed to maternal cigarette smoking
Source: Clin Epigenetics. 2016 Nov 25;8:128. doi: 10.1186/s13148-016-0296-0 (PMC5123323; doi:10.1186/s13148-016-0296-0)
Supplement: Additional file 1: Table S1. — Description of fetal and placenta samples. The number of samples (N) and fetal age in days are illustrated. Samples were assigned to the smoking-exposed group if mothers smoked one or more cigarettes per day (approximately 90% of the smoking mothers were smoking 6–20 cigarettes per day). Exposure to passive smoking was not reported among the non-smoking mothers, whereas among the smoking mothers most also reported exposure to passive smoking. Unpaired t test was used to determine whether there is a statistically significant age difference between the non-smoking and smoking groups and between females and males used for a particular assay (LINE-1, AluYb8, and 5-mC ELISA). *Indicates statistical significance, p < 0.05. (DOCX 17 kb) [file 13148_2016_296_MOESM1_ESM.docx]

**Table 1.** ANCOVA for the effects of fetal age, gender, and PEMCS for DNA methylation levels in first trimester placentas, fetal livers, and small intestines.

| Tissue | Assay | Variable | F-value | P-value |
| --- | --- | --- | --- | --- |
| Placenta | 5mc ElISA | Age | 8.567 | 0.008* |
|  |  | Gender | 8.434 | 0.008* |
|  |  | PEMCS | 0.704 | 0.411 |
|  | *AluYb8* | Age | 0.059 | 0.811 |
|  |  | Gender | 4.527 | 0.042* |
|  |  | PEMCS | 2.958 | 0.096 |
|  | *LINE-1* | Age | 0.376 | 0.544 |
|  |  | Gender | 0.013 | 0.909 |
|  |  | PEMCS | 1.017 | 0.320 |
|  |  |  |  |  |
| Liver | 5mc ElISA | Age | 1.214 | 0.286 |
|  |  | Gender | 0.191 | 0.667 |
|  |  | PEMCS | 0.001 | 0.977 |
|  | *AluYb8* | Age | 3.190 | 0.085 |
|  |  | Gender | 0.004 | 0.953 |
|  |  | PEMCS | 0.106 | 0.747 |
|  | *LINE-1* | Age | 0.263 | 0.612 |
|  |  | Gender | 8.779 | 0.006* |
|  |  | PEMCS | 0.672 | 0.419 |
|  |  |  |  |  |
| Small | 5mc ElISA | Age | 3.576 | 0.078 |
| intestine |  | Gender | 0.445 | 0.515 |
|  |  | PEMCS | 0.147 | 0.707 |
|  | *AluYb8* | Age | 0.006 | 0.942 |
|  |  | Gender | 1.417 | 0.250 |
|  |  | PEMCS | 0.121 | 0.733 |
|  | *LINE-1* | Age | 0.327 | 0.575 |
|  |  | Gender | 0.568 | 0.462 |
|  |  | PEMCS | 1.783 | 0.201 |

Analyses were done with age as a continuous variable and gender and PEMCS as factors. * indicates statistical significance, p<0.05.
