# Supplementary figures and images for: Assessment of global DNA methylation in the first trimester fetal tissues exposed to maternal cigarette smoking
Source: Clin Epigenetics. 2016 Nov 25;8:128. doi: 10.1186/s13148-016-0296-0 (PMC5123323; doi:10.1186/s13148-016-0296-0)

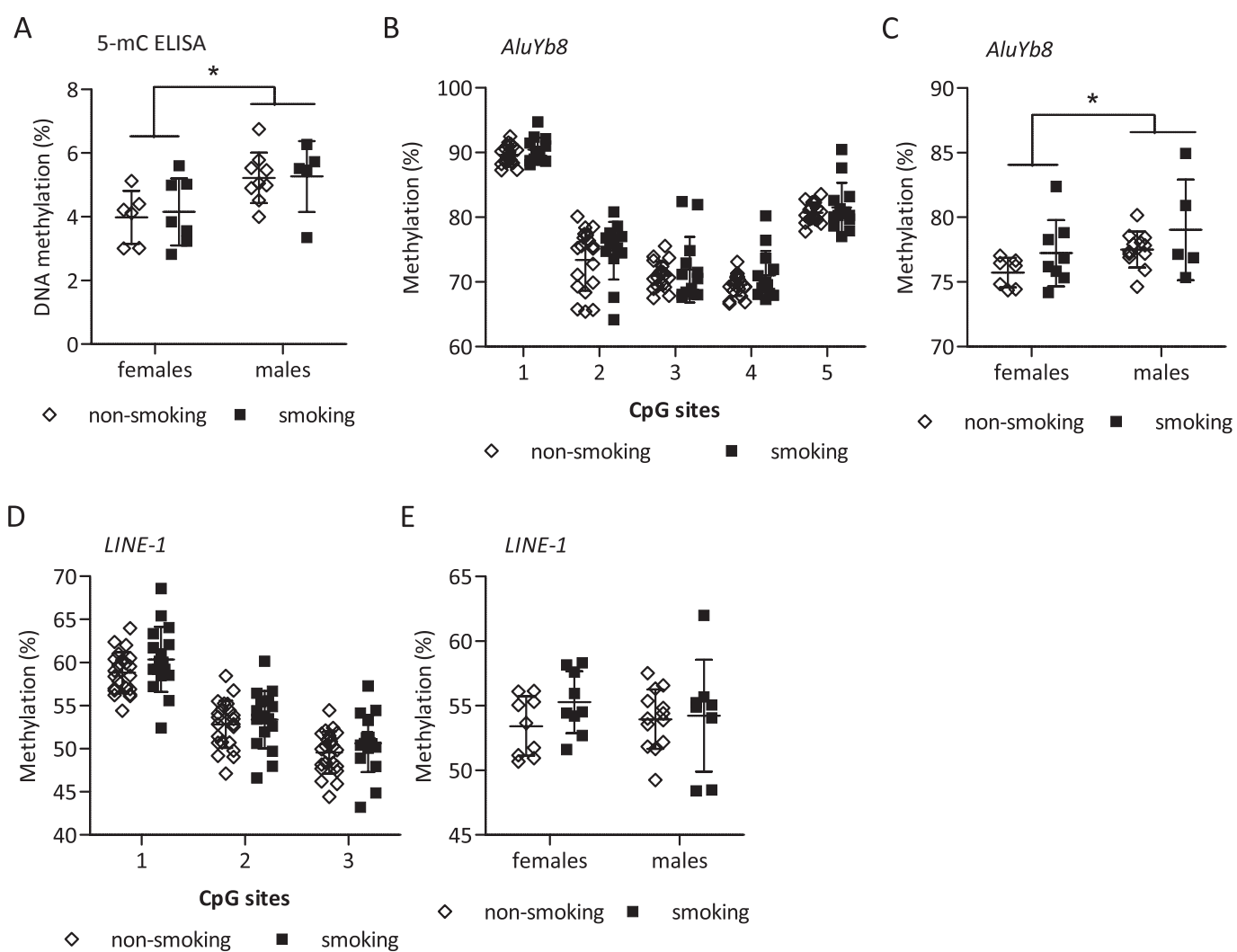

**FIG S1**

Supplement: Additional file 2: Figure S1. — DNA methylation in placentas. a DNA methylation in female and male fetal placentas determined by the Elisa method using 5-mC-specific antibody (non-smoking female group N = 6, non-smoking male group N = 9, smoking female group N = 7, and smoking male group N = 5). Data represent the mean of two experiments. b Percentage of AluYb8 DNA methylation at five consecutive CpG sites (non-smoking group N = 19 and smoking group N = 13). c AluYb8 DNA methylation in males and females (non-smoking female group N = 7, non-smoking male group N = 12, smoking female group N = 8, and smoking male group N = 5). d Percentage of LINE-1 DNA methylation at three consecutive CpG sites (non-smoking group N = 22 and smoking group N = 17). e LINE-1 DNA methylation in males and females (non-smoking female group N = 9, non-smoking male group N = 13, smoking female group N = 9, and smoking male group N = 8). Percentages of methylation were calculated and displayed as described in the legend for Fig. 1. Unpaired t test was used to compare non-smoking and smoking exposed group, while two-way ANOVA was used to compare combined effects of smoke exposure and gender. *Indicates statistical significance, p < 0.05. (PDF 559 kb) [file 13148_2016_296_MOESM2_ESM.pdf]

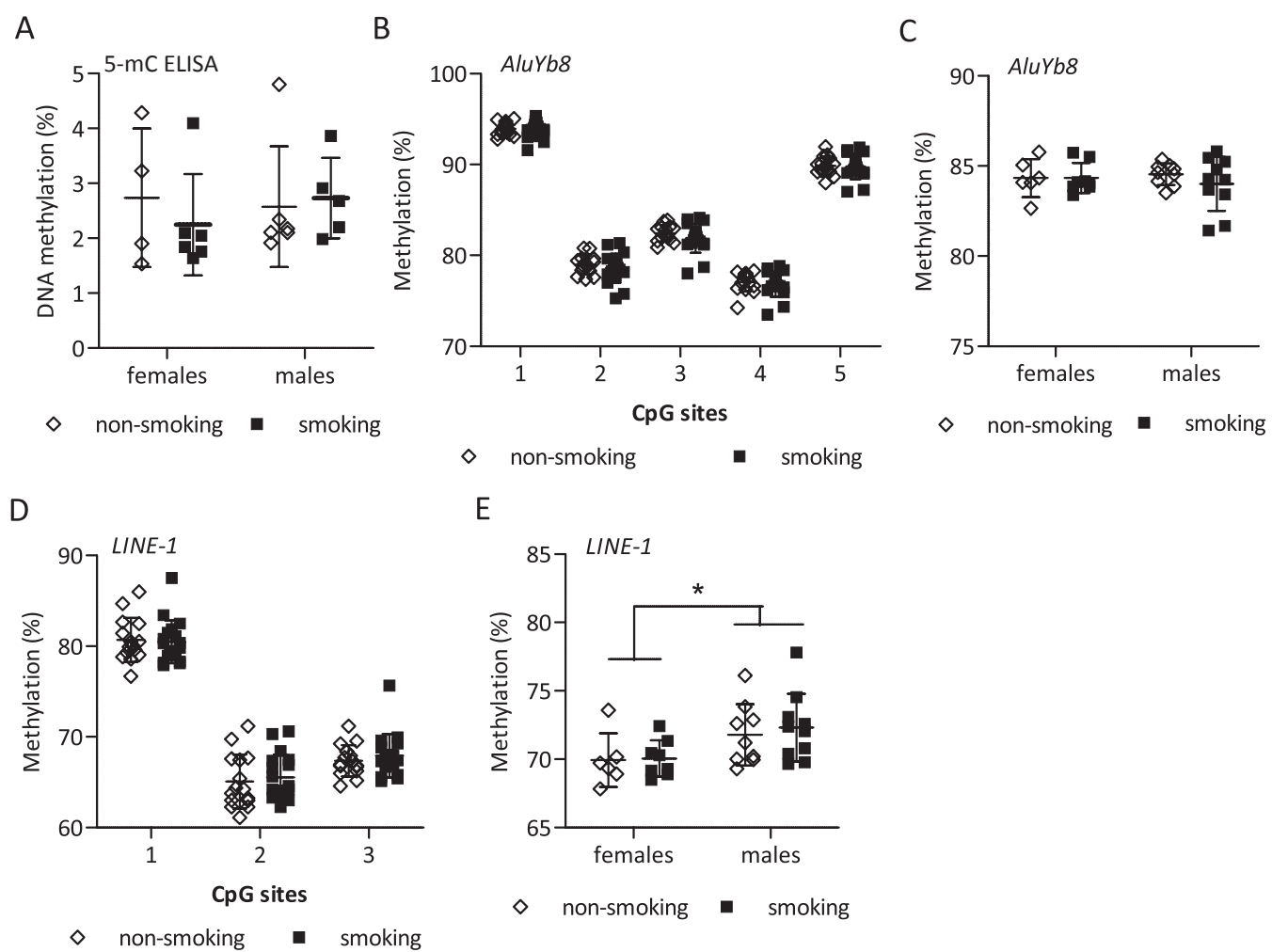

**FIG S2**

Supplement: Additional file 3: Figure S2. — DNA methylation in fetal livers. a DNA methylation in female and male fetal livers determined by the Elisa method using 5-mC-specific antibody (non-smoking female group N = 4, non-smoking male group N = 6, smoking female group N = 6, and smoking male group N = 5). Data represent the mean of three experiments. b Percentage of AluYb8 DNA methylation at 5 consecutive CpG sites (non-smoking group N = 15 and smoking group N = 18). c AluYb8 DNA methylation in males and females (non-smoking female group N = 6, non-smoking male group N = 9, smoking female group N = 8, and smoking male group N = 10). d Percentage of LINE-1 DNA methylation at three consecutive CpG sites (non-smoking group N = 15 and smoking group N = 18). e LINE-1 DNA methylation in males and females (non-smoking female group N = 6, non-smoking male group N = 9, smoking female group N = 8, and smoking male group N = 10). Percentages of methylation were calculated and displayed as described in the legend for Fig. 1. Unpaired t test was used to compare non-smoking and smoking exposed group, while two-way ANOVA was used to compare combined effects of smoke exposure and gender. *Indicates statistical significance, p < 0.05. (PDF 539 kb) [file 13148_2016_296_MOESM3_ESM.pdf]

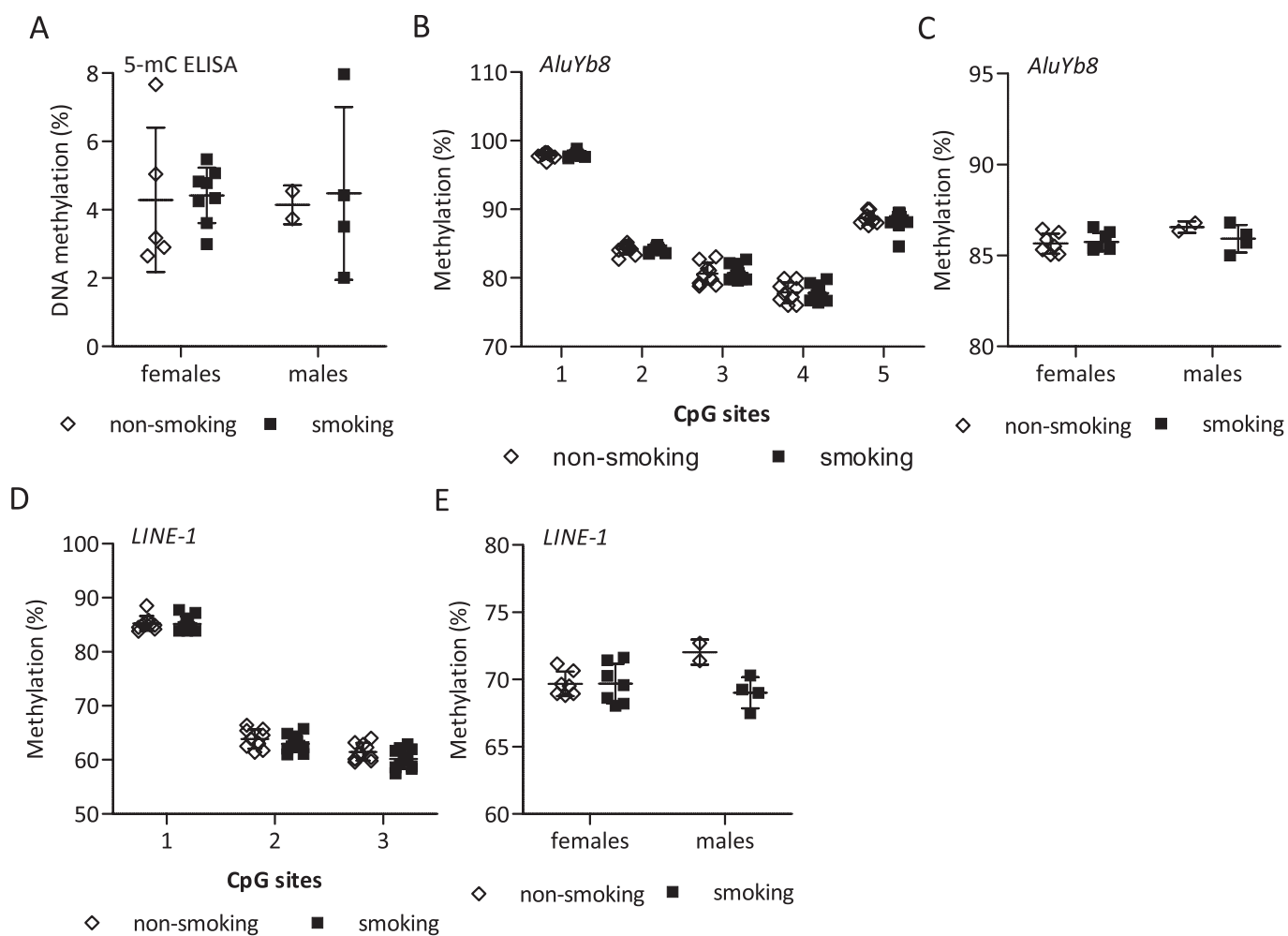

**FIG S3**

Supplement: Additional file 4: Figure S3. — DNA methylation in fetal small intestines. a DNA methylation in female and male fetal small intestines determined by the Elisa method using 5-mC-specific antibody (non-smoking female group N = 5, non-smoking male group N = 2, smoking female group N = 8, and smoking male group N = 4). Data represent the mean of two experiments. b Percentage of AluYb8 methylation at 5 consecutive CpG sites (non-smoking group N = 9 and smoking group N = 12). c AluYb8 DNA methylation in males and females (non-smoking female group N = 7, non-smoking male group N = 2, smoking female group N = 8, and smoking male group N = 4). d Percentage of LINE-1 DNA methylation at three consecutive CpG sites (non-smoking group N = 9 and smoking group N = 11). e LINE-1 DNA methylation in males and females (non-smoking female group N = 7, non-smoking male group N = 2, smoking female group N = 7, and smoking male group N = 4). Percentages of methylation were calculated and displayed as described in the legend for Fig. 1. Unpaired t test was used to compare non-smoking and smoking exposed group, while two-way ANOVA was used to compare combined effects of smoke exposure and gender. (PDF 529 kb) [file 13148_2016_296_MOESM4_ESM.pdf]
